# Respiratory Syncytial Virus Infection: From Biology to Therapy *A Perspective*

**DOI:** 10.1097/WOX.0b013e31816549a2

**Published:** 2008-02-15

**Authors:** Shyam S Mohapatra, Richard F Lockey

**Affiliations:** 1 Joy McCann Culverhouse Airway Disease Research Center, Department of Internal Medicine, University of South Florida College of Medicine; 2 James A. Haley VA Medical Center, Tampa, FL

**Keywords:** respiratory syncytial virus infection, allergic disease, chitosan, nanoparticles

## Abstract

Respiratory syncytial virus (RSV) is responsible for significant morbidity and mortality, particularly in infants younger than 18 months and in the elderly. To date, there are few effective treatment options available to prevent or treat RSV infections. Attractive therapeutic strategies include targeting host epithelial adhesion molecules required for RSV infection, enhancing localized cell-mediated immunity, interfering with RSV viral gene expression and developing a multigene DNA vaccine. The most recent data supporting the advantages and limitations of each of these approaches are discussed in detail. Several promising strategies offer hope for safe and effective prophylaxis and treatment of RSV infection.

## 

Respiratory syncytial virus (RSV) is one of the most important respiratory pathogens targeting all age groups; however, infants (younger than 18 months) and the elderly experience the most severe aspects of the disease, which results in lower respiratory tract illnesses (ie, bronchiolitis and pneumonia) [1]. Around 90% of infants are infected for the first time by the age of 2 years [1,2]. Worldwide, about 5 million infants are hospitalized because of severe RSV infection. The first is usually the most severe, and previous findings indicate that infants with a history of premature birth, bronchopulmonary dysplasia, congenital heart disease, cystic fibrosis, or immunosuppression are more likely to develop the most severe clinical courses of bronchiolitis and pneumonia, which have the highest risk of death[1,2].

However, an analysis of a comprehensive study done between 1979 and 1997 about RSV-associated deaths in US children suggests that most RSV-related deaths do not occur among children who are presumed to be at high risk for severe RSV lower respiratory tract illnesses [3]. The leading cause in infant hospitalization is RSV bronchiolitis, [4] which imposes a severe burden upon health services. Costs related to emergency department visits between 1997 and 2000 amount to approximately 202 million US dollars [4]. Complete immunity to RSV never develops, and reinfection throughout life is common. Although the major clinical manifestation of RSV in older children and adults is upper respiratory tract illness (rhinitis and acute otitis media), it may also cause up to 2.4% of community-acquired pneumonia in these population groups [5]. In older adults, RSV was identified as responsible for 10% of winter hospital admissions and has a case-fatality rate that approaches 10%. In addition, 78% of RSV-associated deaths occur in individuals aged 65 years or older who have underlying cardiac and pulmonary pathology [6]. In particular, RSV infection in adults with strong immunosuppression, for example, patients undergoing bone marrow transplantation is of great medical importance [7].

In the past 8 years, our research has identified both cellular and viral targets that may be useful for the prevention of RSV infection and its accompanying pathology. Differential microarray analysis was used to pinpoint gene expression changes in RSV-infected cells, and expression of candidate therapeutic genes was tested both in cultured lung epithelial cells in vitro and in animal models in vivo. Characterization of these gene expression changes includes immune modulation, signal transduction, and apoptosis. In this report, the biology of RSV and how these studies contribute to the basic mechanistic studies of RSV infection and have led to new targets to manage RSV infection will be discussed.

## State of the Art in Treatment and Prophylaxis of RSV Infection

There is no treatment to protect against RSV infection, and the current treatment, Ribavirin, only produces modest short-term improvement in respiratory tract infection [8]. Moreover, it is now restricted to a highly selected group of patients with T-cell immunodeficiency [9]. Passive immunoprophylaxis, involving the administration of either a polyclonal antibody (Synagis) preparation or a humanized version of a monoclonal anti-RSV-F antibody (Palivizumab), is successful for protection of high-risk individuals against RSV infection. However, these approaches are only partially effective, expensive, and could generate resistant mutant RSV strains. Development of new and highly effective antibodies to modulate RSV infection remains a major medical and pharmaceutical goal.

To date, there is no licensed vaccine for the prevention of human RSV disease. Efforts have been made to develop active prophylaxis measures (vaccines), and both subunit and attenuated live vaccines are being pursed in clinical studies. Vaccine development has been limited after the testing of initial vaccines in the 1960s, which exacerbated the RSV disease [10,11]. Some of the reasons for the lack of success in developing previous vaccines include the inadequate response to vaccination, the existence of 2 antigenically distinct RSV groups, and the history of disease enhancement after administration of a formalin-inactivated vaccine [12,13].

Developing active or passive prophylaxis is important as they are expected to decrease the incidence of severe infections and thus may reduce or attenuate asthma pathogenesis. Recent advances in the vaccine area include research with plasmid-based DNA vaccines and small-interfering RNA (siRNA)-based approaches. To deliver these antiviral plasmids in the most effective way to target cells, a novel carrier system has been produced based on modified polysaccharide nanoparticles that protect the DNA and facilitate its introduction into the lungs. The advances in this field are reviewed in the following sections.

## RSV Genome and Structure

Human RSV is in the genus *Pneumovirus*, subfamily *Pneumovirinae*, family *Paramyxoviridae*, order *Mononegavirales*, whose members consist of nonsegmented, negative-sense, single-stranded RNA viruses. In addition to human RSV, the genus *Pneumovirus *includes bovine RSV, ovine RSV, and pneumonia virus of mice. The RSV virions consist of a nucleocapsid contained within a lipid envelope of irregular spherical shape with sizes of 150 to 300 nm. Both infected cultures and viral preparations can also include filamentous forms of the virions that are 60 to 100 nm in diameter and up to 10 μm in length [14]. The viral envelope is a lipid bilayer acquired from the host plasmatic membrane. The viral transmembrane glycoproteins--the fusion protein F, the attachment protein G, and the small hydrophobic protein SH--organize themselves to form spikes, which are visible under electron microscopy. Host lipid raft-derived proteins are also incorporated into the envelope of mature viral particles [15-17]. The envelope connects to the nucleocapsid through the viral matrix M protein. Using electron microscopy, the nucleocapsid is seen as an internal electrodense material with a diameter of 15 nm inside the round and filamentous forms of the virions [14]. The nucleocapsids consist of the RNA genome and the associated nucleocapsid protein N, the phosphoprotein P, the large polymerase subunit L, and the antitermination factor M2-1. The viral RNA genome and the associated proteins in the nucleocapsid together form a very tight ribonucleoprotein complex, which is resistant to RNAse activity.

The genome for most of the virions is a negative-sense strand of RNA of 15,222 nucleotides in length. However, some virions are also found to have incorporated the positive-sense replicative intermediate (antigenomic RNA), which is synthesized during viral replication. Thus, this implies that during the viral assembly, there is no mechanism that allows discrimination in packaging. The viral genes are ordered from 3' to 5' in the following way: NS1-NS2-N-P-M-SH-G-F-M2-L. Glycoprotein G and F (and SH), respectively, mediate virus attachment and fusion to the host cell [18]. In addition to fusion, protein F has also been postulated to participate in the attachment of the virus to the host cell membrane. Intercellular adhesion molecule 1 (ICAM-1), annexin-II, and Toll-like receptor 4 are receptors for protein F [19-21]. The matrix (M) protein forms a layer on the inner face of the viral envelope, and it plays an essential role in viral assembly through its interactions with the cell membrane, virus envelope, and virus nucleocapsid [22,23]. The nucleocapsid-associated proteins N, P, M2-1, and L play essential roles at different stages for efficient viral transcription and replication. The nonstructural proteins NS1 and NS2 are thought to be antagonists of the interferon (IFN)-type I system. They seem to target the transcription factor IRF-3. Thus, the expression of these proteins helps the virus to reduce IFN-γ expression by infected cells [24,25].

## Prophylaxis and Treatment of RSV Infection

**Figure 1:**
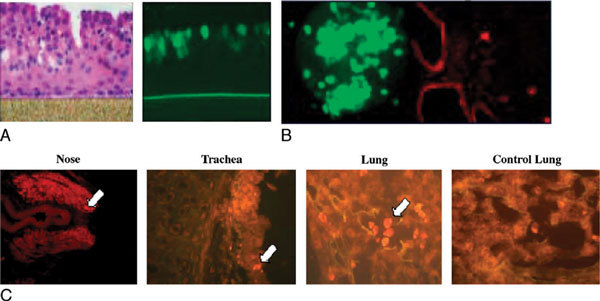
**Model systems for RSV infection**. A, The RSV infection is studied in human 3-dimensional epithelial cell cultures. Left panel, Hematoxylin and eosin staining of cells. Right panel, Infection of ciliated epithelial cells by green fluorescent protein-RSV. B, Left panel, Infection of human dendritic cells by green fluorescent protein-RSV. Right panel, Infection of mouse epithelium and dendritic cells by rhodamine-labeled anti-RSV antibodies. C, Immunohistochemical analyses of section from mouse nose, trachea, and lung, and localization of RSV infection.

Developing antivirals requires a comprehensive molecular understanding of the early events of virus-host interaction necessary for viral fusion and entry into cells and viral replication. To study viral interactions, human epithelial cell cultures, a 3-dimensional epithelium, and human dendritic cell and mouse models of RSV infection have been established in our laboratory (Figures 1A-C). The RSV affects pulmonary function in BALB/c mice [26]. A number of investigators have used a mouse model for the study of asthma and RSV infection using an inbred BALB/c strain of mouse [27-32]. Figure 1C shows the localization of RSV in the nose, trachea, and lung of BALB/c mice after their infection with RSV by immunohistochemical analyses. The sections stained for RSV were produced from mouse nose after 1 hour of RSV infection. The negative controls did not exhibit any RSV specific staining (red). One side of the nose of infected mice showed RSV, also the tracheal epithelium and peripheral lung sections showed RSV infection. Macrophages were infected with RSV in the peripheral lung. No infection was found in the control mice. As in humans, pulmonary T cells induce both T_h_1 and T_h_2 responses in the lung in response to RSV infection [31-35]. The contributions of our laboratory fields are summarized in Table 1.

**Table 1:** Summary of Studies Relating to RSV Infection

Area	Protein(s) Involved	Therapeutic Approach	Reference
Host	ICAM-1	Ab, antisense RNA	[87-89]
proteins	IFN-γ	Gene therapy	[48,90,91]
	2-5'Oligoadenylate synthetase	Gene therapy	[92]
	ERK1, 2	SMD inhibitors	[93]
	STAT-1/3	SMD inhibitors	[94]
	PKC-α	SMD inhibitors	[95]
Virus	F	Antisense RNA	
proteins	NS1	siRNA	[96]
	F, G, SH, NS1, NS2, P, N	DNA vaccine	[90,97]

ERK indicates extracellular signal-regulated kinase; PKC, protein kinase C; SMD, small molecular drug; STAT, signal transduction and activator of transcription.

**Figure 2:**
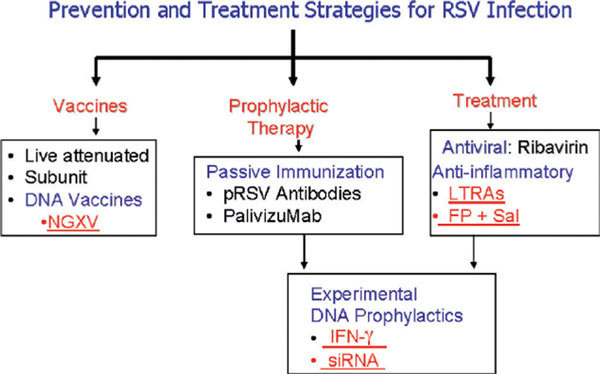
**A schematic diagram of the methods under the current research for the prevention, prophylaxis, and treatment of RSV infection**. Underlined areas represent contributions reported in this article.

Similarly, the methods of prevention and treatment are shown in Figure 2. The salient findings thus far are as follows: (1) RSV infection induces the expression of ICAM-1 on host cells. The colocalization of RSV and ICAM-1 suggests that ICAM-1 binds to RSV, most likely by interacting with the RSV fusion protein. Treatment of cells with antibodies to ICAM-1 or targeting ICAM-1 in mice significantly inhibits RSV infection and the production of inflammatory mediators, suggesting a therapeutic potential of anti-ICAM-1 approaches; (2) intranasal administration in mice of a plasmid encoding IFN-γ significantly decreases viral replication in the mouse lung and reduces lung inflammation. From DNA microarray analysis and other molecular and cellular techniques, we have identified 2-5 antisense oligoadenylate synthetase as an important molecule in the IFN-γ-mediated inhibition of RSV replication. Mice given adenovirus expressing 2-5 antisense oligoadenylate synthetase significantly inhibit RSV replication; (3) from microarray studies to dissect the early events of RSV infection, multiple signaling pathways involving STAT1 and STAT3, ERK-1 and ERK-2, and PKC-α are involved in RSV-induced early gene expression and inflammation. PKC-α is a critical target upstream of these signaling pathways, and inhibitors of PKC-α specifically block RSV fusion and stop the infection of normal human bronchial epithelial cells. To elucidate the mechanism of RSV infection, RSV-induced signal transduction pathways involving STAT and PKC were investigated. These studies in human epithelial cells have now been extended to RSV-infected mouse model, where it has been possible to localize up-regulation of multiple signaling pathways such as those involving nuclear factor-κB (NF-κB) has been localized to infected lung cells (Figure 2); (4) finally, to develop a vaccine for prophylaxis or treatment based on RSV genes, a multigene DNA vaccine and siRNA-based strategy was explored. The contributions to the development of a nanotechnology platform for a DNA vaccine and for RNA interference therapy are summarized in the following sections.

### Development of Chitosan-Based Nanoparticles as a Platform for Gene and Drug Delivery

Numerous investigators, including those in our laboratory, have extensively studied chitosan, which we believe has the potential to be useful for the delivery of genes and drugs, as it has very low immunogenicity while having strong immunostimulatory properties [36]. Moreover, as a carrier, it can most adequately provide heat stability to encapsulated or adsorbed vaccines. Chitosan, a natural biocompatible cationic polysaccharide extracted from crustacean shells, is capable of efficient drug and gene delivery [37-41]. Chitosan has many beneficial effects, including anticoagulant activity, [36] wound-healing properties, [42] and antimicrobial properties [42]. In addition, chitosan is nontoxic, nonhemolytic, slowly biodegradable, and nuclease resistant, and it has been widely used in controlled drug delivery [37,43-47]. Chitosan also increases transcellular and paracellular transport across the mucosal epithelium[48] and, thus, may facilitate mucosal drug delivery and modulate immunity of the mucosal and bronchus-associated lymphoid tissues. Chitosan apparently binds to macrophages and myeloid cells via CD14.[49,50]

The toxicity of mucosally administered chitosan has been studied in rodents. *N*-trimethyl chitosan and chitosan hydrochloride given intranasally do not alter the ciliary beat frequency of the rat nasal epithelium, and hence, both are considered to be nontoxic [51]. In addition, the subacute oral toxicity of chitosan oligosaccharides was investigated in Sprague-Dawley rats of both sexes [52]. The chitosan is metabolized and secreted through the viliary system. Thirty-six male and female rats were administered by gavage 500, 1000, and 2000 mg/kg per day of chitosan for 4 weeks (7 days per week), and their clinical signs, body weights, hematologic and biochemical parameters, and histopathology were examined. There were no significant differences in behavior, external appearance, body weight or food consumption between control and treated rats. In addition, no significant differences in urinalysis, hematology, blood biochemistry, relative organ weights, and histopathological findings were found in either control or treated rats. These results suggest that the acute toxicity of chitosan oligosaccharides is low and that the detection limit of toxicity is greater than 2000 mg/kg in rats. Furthermore, chlorophyllin-chitosan, an insoluble form of chlorophyllin, inhibits DNA adduct formation and mutagenesis by a heterocyclic food mutagen-carcinogen, 3-amino-1-methyl-5H-pyridoindole (Trp-P-2), in mice carrying the *Escherichia coli *rpsL gene as a mutagenesis reporter, this suggests that chlorophyllin-chitosan may be a candidate chemopreventive agent against the genotoxic action of Trp-P-2 and possibly other aromatic carcinogens in the diet.[53]

The Environmental Protection Agency has ruled chitosan exempt from its tolerance guidelines because of its nontoxicity as evidenced by the: (1) literature search done for chitin, chitosan, *N*-acetyl-D-glucosamine, and D-glucosamine toxicity in humans using the databases PubMed, Hazardous Substances Data Bank, Integrated Risk Information System, Gene-Tox, Environmental Mutagen Information Center, Toxic Release Inventory, the Food and Drug Administration, the United States Department of Agriculture and ChemIDplus; (2) animal feeding studies, in which up to 5% of the diet is chitosan, that failed to show any adverse effects; and (3) the lack of reported complaints of toxicity against the database of 2700 complaints despite years of chitosan use in food and nutritional supplements.

**Figure 3:**
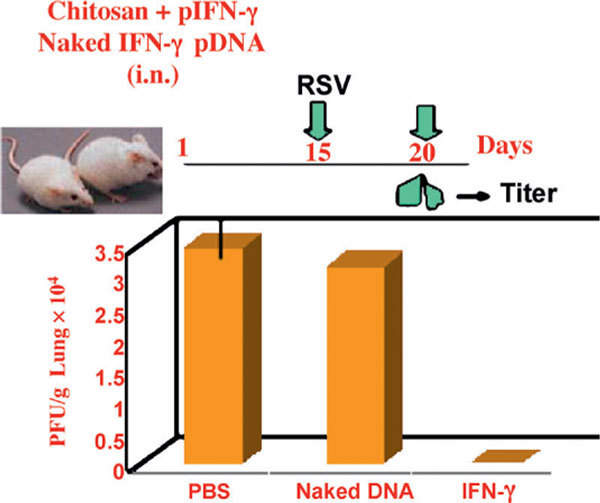
**Anti-RSV activity of pIFN-γ nanoparticles in mice**. Nanoparticles complexed with plasmid encoding IFN-γ (pIFN-γ) decreased lung RSV titers. i.n. indicates intranasal; PBS, phosphate buffered saline.

Several double-blind placebo-controlled human studies demonstrate the safety of chitosan when given orally. The results of these studies show chitosan-mediated decreases in total cholesterol level, [53-55] decreases in serum low-density lipoprotein cholesterol, [56] increases in fetal fat excretion, [57] and increases in vitamin K [58]. No chitosan-mediated reductions in body weight were observed [56,58]. Chitosan was tolerated, and no serious adverse events or changes in safety parameters were noted, including serum levels of fat-soluble vitamins A, D, E, and Fe++ and transferrin [56]. Given these numerous reports on safety and lack of toxicity, chitosan-based nanoparticles provide a great opportunity to deliver proteins, peptides, drugs, and genes. Furthermore, a number of investigators have taken advantage of the cationic property of chitosan and used chitosan for targeted delivery of drugs and other biologics via the mucosal route, maximizing the drug effectiveness and minimizing the adverse effects by slow sustained release of the drug (Figure 3). Thus, the advantages of chitosan nanoparticles as a platform for vaccine or therapy are: (1) ease of construction of DNA-based constructs, (2) stability and heat resistance, (3) ease of use and preparation, (4) possibility to use cocktails, (5) lack of replication in mammalian cells, (6) lack of integration into host genomes, (7) the possibility for persistent expression, and (8) expression of the cloned gene for a period of weeks to months. We have used this platform for an intranasal gene expression vaccine, for the expression of cytokine IFN-γ as a prophylactic/treatment, and for delivery of RNA interference therapy based on a nonstructural gene, NS1, of RSV.

### A Nanoparticle Gene Expression Vaccine for RSV

The potential of vaccines has been intensely investigated since the discovery of the virus. All RSV proteins, except L, have been tested for immunogenicity and protective efficacy in rodents using recombinant vaccinia viruses [59-61]. A number of approaches, including recombinant live, attenuated, subunit vaccines, and DNA vaccines, are under intense investigation, [62-64] but none have crossed the clinical-phase hurdles and been licensed thus far. The development of RSV vaccines is complicated by the need to administer the vaccine at a very young age, between 6 weeks and 6 months, in the face of a premature immune system. In addition, because RSV is a mucosal pathogen, an effective vaccine must generate secreted mucosal antibodies, such as immunoglobulin A (IgA) and mucosal cytotoxic lymphocytes (CTLs) [65,66]. The RSV-induced CTL response at mucosal sites is inadequate. Although evidence suggests the potential of a gene expression vaccine for RSV infection, the number of studies is limited. Previous reports using systemic injections of pDNA show variable results. The quantity of DNA used per unit body mass, as much as 10 mg/kg, and the route of administration chosen are inconvenient for infants and are suboptimal for inducing mucosal immunity against a pulmonary infection.[67]

Our laboratory developed a nanoparticle multigene vaccination strategy against RSV infection using a complementary DNA cocktail produced by cloning 9 RSV antigens (NS1, NS2, M, SH, F, M2, N, G, or P in a pVAX plasmid) complexed with chitosan nanoparticles, referred to as nanoparticle gene expression vaccine (NGXV). The NGXV was administered to mice by the intranasal route. The rationale for developing this vaccine is based on the following reports. All of the RSV proteins, except L, have been tested individually (and in some cases, in combination) for immunogenicity and protective efficacy in rodents using recombinant vaccinia viruses [59-63]. The F and G proteins are the antigens that induce most of the the neutralizing antibodies against RSV [68-70]. The CTL repertoire in humans revealed that the N, SH, F, M, M2, and NS2 proteins were strong target antigens. In BALB/c mice, the F, N, and M2 proteins are major target antigens [61,71-73]. Protection against and recovery from RSV infection are mediated largely by the immune system, with the specific direct effectors being secretory antibodies, serum antibodies, and major histocompatibility complex class I-restricted CTLs.

The results demonstrate that a single vaccination of about 1 mg/kg body weight of NGXV decreases viral titers by 2 orders of magnitude (100-fold) upon primary infection. In addition, NGXV significantly decreases pulmonary inflammation and does not alter airway hyperresponsiveness, thus making it a potentially safe vaccine. This may represent a major breakthrough in RSV vaccine development.

**Figure 4:**
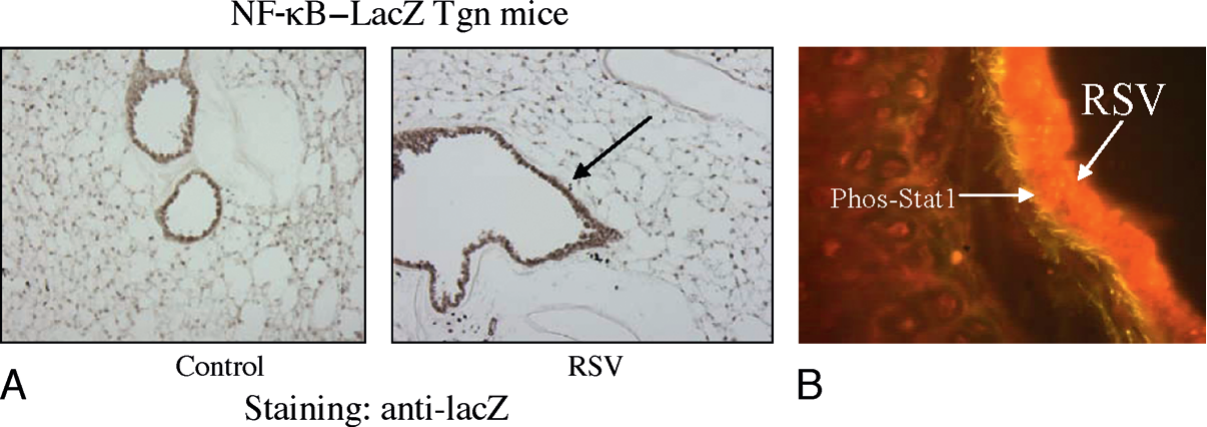
**Immunohistochemical analyses of sections from mouse trachea and localization of NF-κB (lacZ) expression in NF-κB-lacZ Tgn mice**. A, The cryosections stained with phospho-STAT (green) antibody were produced from mice after 1 hour of RSV infection. The negative controls did not exhibit any RSV-specific staining (red). B, Sections were stained with antibody to RSV (red) and lacZ (green). Tgn indicates transgenic.

The immunologic mechanisms for the effectiveness of this vaccine include the induction of both high levels of serum IgG and mucosal IgA antibodies, the generation of an effective CTL response, and elevated lung-specific production of IFN-γ with antiviral action (Figure 4). Although a single dose of NGXV is effective, it is possible that dose escalation and prime-booster strategies might further enhance its effectiveness.

### Immunoprophylaxis

#### Host Gene Expression

Prophylactic IFN-γ gene transfer in BALB/c mice decreases viral replication and induces a T_h_1-like (increased production of IFN-γ and interleukin-12), instead of a T_h_2-like (decreased interleukin-5) immune response against RSV infection [74-76]. Viral infections induce IFN-γ, which in turn facilitates the resolution of viral infection [74]. Levels of IFN-γ have been compared in bronchoalveolar lavage fluids after infection with RSV in control and pIFN-γ-treated mice. A 3-to 6-fold increase in IFN-γ production was found in RSV-infected mice compared with uninfected mice. Such increases are considered to be relatively low compared with other viral infections [74-76]. The finding that a natural live virus infection is cleared by elevated IFN-γ production, a response similar to that seen after live viral infection in mice, suggests that the results from this animal model may be applicable to human RSV disease.

### SiRNA-Based Prophylaxis

A new prophylactic approach consists of taking advantage of the RNA interference mechanism initially discovered in plant cells and that is present in all species including mammals. RNA interference is triggered by double-stranded RNA that is cleaved by an RNAse III-like enzyme, Dicer, into 21-25-nucleotide fragments (siRNAs) with characteristic 5' and 3' termini [77,78]. These siRNAs act as guides for a multiprotein complex, including a PAZ/PIWI domain, containing the protein Argonaute2, which cleaves the target messenger RNA (mRNA) [79]. These gene-silencing mechanisms are highly specific and induce inhibition of gene expression throughout an organism. RNA interference is a known phenomenon that has been proven effective in silencing a number of genes of different viruses [80-82]. The siRNA to viral P or NS-1 mRNAs prevents RSV infection in cellular and animal model studies [83,84]. Prophylactic intranasal administration of an siRNA formulation specific for RSV-P mRNA is able to significantly reduce the viral load and the disease parameters in RSV-infected mice [83]. A carrier in the formulation is not required. In addition, a very low dose is effective in showing a protective effect. Moreover, siRNA-resistant virus did not appear after using this formulation [83]. Although intranasal administration of naked siRNA to humans was found to be safe in a phase I study, other studies have shown toxicity.

Because the synthesis of RNA oligonucleotide-based siRNA is expensive, our laboratory engineered DNAvector-based approaches to introduce siNS1 into RSV-infected human cells and animal models. This is based on the principle of the intracellular transcription of small RNA molecules that are synthesized from a DNA template under the control of RNA polymerase III promoters, such as U6 [85]. NS-1 was selected as the target because the NS1 protein interferes with type-1 IFN-mediated host antiviral responses [24,86]. Silencing of the NS-1 gene attenuated RSV replication and boosted the immune response through an increase in IFN-γ production [84]. The prophylactic intranasal administration of this formulation, combined with chitosan, significantly reduced the viral load and ameliorated the pulmonary pathology in RSV-infected mice [84]. In addition, mice treated with this formulation develop protection against reinfection.[84]

Moreover, this formulation also drives human dendritic cells to promote a T_h_1-like profile [84]. Overall, siRNA-mediated silencing of the NS1 gene up-regulates host-antiviral genes and suppresses RSV replication compared with control groups. Studies confirm the role of siNS1 in a rat model of RSV infection. A phase I study is currently under development using the nanoparticle-incorporated siNS1, and it may represent a novel prophylaxis/therapy that can be used in a global population.

## Summary and Conclusion

The RSV is the major pathogen responsible for serious upper and lower respiratory tract infections, primarily in infants, but also in the elderly worldwide. The precise molecular and cellular mechanisms are unclear, and satisfactory prophylaxis or treatment strategies are yet to emerge. This research has resulted in the understanding of the pathology and complexity of signaling pathways involved in successful infection; the role of host defense molecules such as ICAM-1, IFN-γ, and related pathways; and how they can be exploited to develop less costly prophylaxis and treatments for RSV infection. Finally, the potential to develop safe and effective prophylaxis and/or treatment by targeting important RSV genes is under investigation.
